# A Germline Polymorphism of Thymine DNA Glycosylase Induces Genomic Instability and Cellular Transformation

**DOI:** 10.1371/journal.pgen.1004753

**Published:** 2014-11-06

**Authors:** Ashley Sjolund, Antonia A. Nemec, Nicolas Paquet, Aishwarya Prakash, Patrick Sung, Sylvie Doublié, Joann B. Sweasy

**Affiliations:** 1 Department of Genetics, Yale University School of Medicine, New Haven, Connecticut, United States of America; 2 Department of Therapeutic Radiology, Yale University School of Medicine, New Haven, Connecticut, United States of America; 3 Department of Molecular Biophysics and Biochemistry, Yale University School of Medicine, New Haven, Connecticut, United States of America; 4 Department of Microbiology and Molecular Genetics, University of Vermont, Burlington, Vermont, United States of America; University of Washington School of Medicine, United States of America

## Abstract

Thymine DNA glycosylase (TDG) functions in base excision repair, a DNA repair pathway that acts in a lesion-specific manner to correct individual damaged or altered bases. TDG preferentially catalyzes the removal of thymine and uracil paired with guanine, and is also active on 5-fluorouracil (5-FU) paired with adenine or guanine. The rs4135113 single nucleotide polymorphism (SNP) of TDG is found in 10% of the global population. This coding SNP results in the alteration of Gly199 to Ser. Gly199 is part of a loop responsible for stabilizing the flipped abasic nucleotide in the active site pocket. Biochemical analyses indicate that G199S exhibits tighter binding to both its substrate and abasic product. The persistent accumulation of abasic sites in cells expressing G199S leads to the induction of double-strand breaks (DSBs). Cells expressing the G199S variant also activate a DNA damage response. When expressed in cells, G199S induces genomic instability and cellular transformation. Together, these results suggest that individuals harboring the G199S variant may have increased risk for developing cancer.

## Author Summary

DNA repair is vital to the survival and propagation of cells. It helps protect DNA from becoming permanently damaged and prevents cells from becoming cancerous. The base excision repair (BER) pathway is responsible for the removal of up to 20,000 lesions/cell/day. Thymine DNA glycosylase (TDG) is one of the DNA glycosylases that initiates BER. There is a germline variant of TDG that is found in 10% of the global population, where amino acid residue glycine 199 is mutated to serine. Here, we provide evidence that TDG variant G199S binds significantly more tightly to its abasic product and leads to increased DNA strand breaks in cells. We go on to show that G199S induces genomic instability, in the form of chromosomal aberrations, and leads to cellular transformation, both hallmarks of tumorigenesis. Collectively, our work suggests that a germline variant of TDG can drive carcinogenesis.

## Introduction

Thymine DNA glycosylase (TDG) is a monofunctional DNA glycosylase that functions in base excision repair (BER), the pathway responsible for repairing up to 20,000 endogenous lesions/cell/day [1]. This glycosylase is well known for its ability to remove T from G:T mispairs and can also excise a variety of other bases, some of which include U opposite A and 5-fluorouracil (5-FU) paired with A or G [2]–[5]. More recent work has implicated TDG in an active demethylation pathway with the ten-eleven translocation (TET) protein family [6]. It has been shown both biochemically and biologically that TDG can remove TET-generated 5-formylcytosine (5-fC) and 5-carboxylcytosine (5-caC) in a process that requires BER to regenerate unmodified C [6]–[9]. Because both DNA repair and DNA methylation dynamics are vital processes to the maintenance of genomic stability, aberrant activity of either of these processes could contribute to cancer development.

The rs4135113 single nucleotide polymorphism (SNP) of TDG, where G is mutated to A at position 818, has a minor allele frequency of approximately 10%, is most commonly found in African and East Asian populations and is usually heterozygous (www.ncbi.nlm.nih.gov/SNP/). This mutation leads to the substitution of glycine to serine at amino acid residue 199. G199 is located in a loop that appears to stabilize the flipped out abasic site within the active site pocket of TDG and serves to prevent flipping of the residue back into the helix [10]. We hypothesize that mutating this residue could affect the stability of the everted abasic site in the active site pocket.

 There is limited available evidence on TDG polymorphisms in relation to cancer or biomarkers of cancer. One study found G199S to be associated with increased likelihood of micronuclei in Chinese workers who had been exposed to vinyl chloride, suggesting individuals carrying G199S are more susceptible to chromosomal damage [11]. Other studies have found that G199S is not associated with esophageal squamous cell carcinoma or gastric adenocarcinoma in a Chinese population [12], and is not associated with increased risk of non-melanoma skin cancer, lung and rectal cancer [13], [14]. More recent work found that TDG expression levels are upregulated in colorectal carcinoma (CRC) and that TDG serves to regulate Wnt signaling, a key driver for CRC [15]. Interestingly, depletion of TDG significantly inhibited cancer cell proliferation and tumor formation in this study, suggesting TDG is required for CRC growth and may serve as a biomarker. In this study, we tested the hypothesis that expression of G199S has as functional phenotype that could induce cellular transformation.

We report herein that while the purified G199S protein has a glycosylase activity similar to WT, it binds significantly more tightly to its abasic product. Expression of G199S in human breast epithelial cells results in the accumulation of DNA double-strand breaks and activates a DNA damage response in cells progressing through the S- and G2/M-phases of the cell cycle. Expression of G199S also leads to the induction of chromosomal aberrations and cellular transformation. Our results suggest that carriers of the rs4135113 SNP may have an increased cancer predisposition.

## Results

### Germline variant G199S has similar catalytic activity to the WT protein

**Figure 1:**
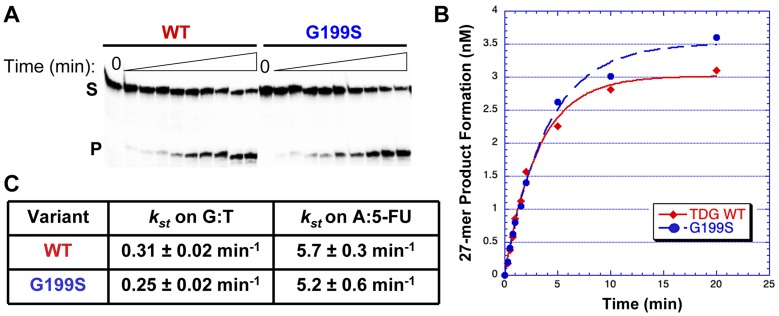
G199S has similar catalytic activity to WT TDG. Equal concentrations of WT (*filled diamonds*) or G199S (*closed circles*) protein (75 nM) were incubated with 5 nM of ^32^P-labeled oligonucleotides (Table 1) containing a G:T or A:5-FU mispair for up to 20 mins at 37°C. Reactions were terminated with NaOH, heated and mixed with formamide loading dye. A and B. Representative data with the G:T substrate. A. The first lane is a no protein control while the following lanes have protein with increasing incubation time up to 20 mins. S represents substrate; P represents product. B. Data were plotted as product formed *versus* time and fit to a single exponential equation. C. Catalytic rates (*k_st_*) for WT and G199S on G:T and A:5-FU substrates.

The rs4135113 SNP encodes the G199S variant TDG protein. To determine if alteration of amino acid residue Gly199 to Ser affects the catalytic activity of the protein, we purified the protein and performed glycosylase assays as described in Materials and Methods. We characterized TDG glycosylase activity using a radiolabeled DNA substrate containing either a G:T mispair or a 5-FU lesion opposite A under single turnover conditions, where protein is in excess of DNA (Table 1). Under these conditions, product release and inhibition do not affect the observed rate. The data were fitted to a single exponential of product formation, typical of TDG activity, and the catalytic rates (*k_single turnover_*) were derived (**Figure 1**). We found that WT and G199S TDG had similar catalytic rates on both G:T (*k_st_* = 0.31±0.02 min^−1^ and 0.25±0.02 min^−1^, WT and G199S, respectively) and A:5-FU substrates (*k_st_* = 5.7±0.3 min^−1^ and 5.2±0.6 min^−1^, WT and G199S, respectively). These data indicate that mutating Gly199 to serine does not affect the glycosylase activity of TDG.

**Table 1:** DNA substrates.

DNA Substrate	Sequence	Assay
38-mer	5′-CAATCCTAGCTGACACGATGTGGCCAATGGCATGACTC-3′	Single Turnover Kinetics
	3′-GTTAGGATCGACTGTGXTACACCGGTTACCGTACTGAG-5′	
	X = T	
28-mer	5′-CAGCTCTGTACATGAGCAGTGGTGACAC-3′	Gel Mobility Shift Assay and Single Turnover
	3′-GTCGAGACATGXACTCGTCACCACTGTG-5′	
	X = 5-FU and THF	

### Germline variant G199S binds significantly more tightly to its abasic product

**Figure 2:**
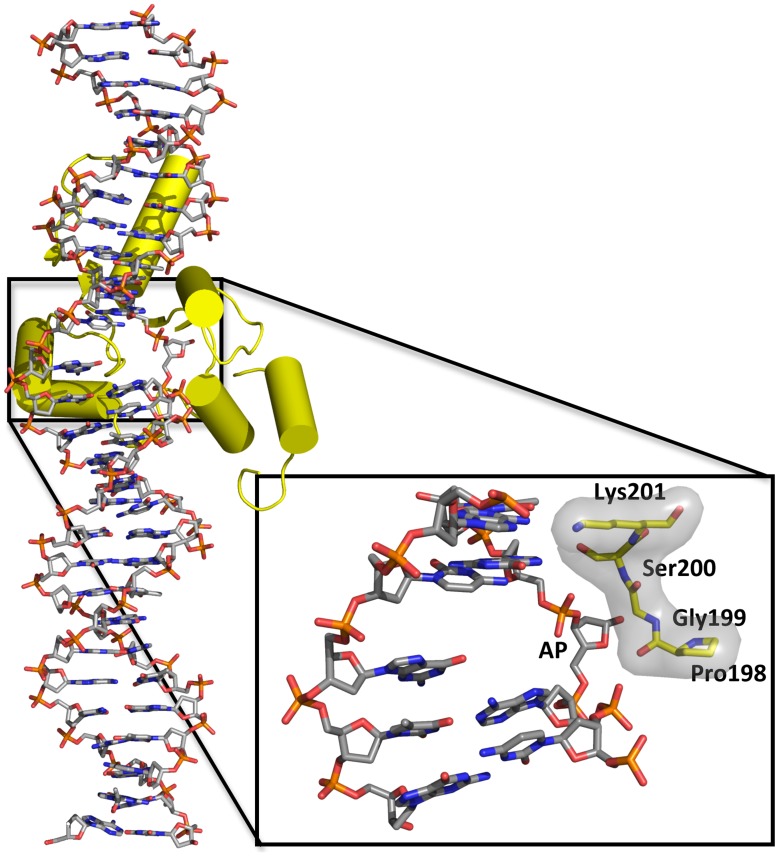
Overall structure of the complex of human TDG with DNA containing a G:5hmU mismatch (PDBID code 4FNC; [9]) and close-up of the resulting abasic site (AP) everted from the double helix. TDG (yellow) is shown as a cartoon representation and the DNA as a stick model. As shown in the magnified part of the active site, Gly199 is located in a loop that makes a close approach to the everted region of the DNA.

**Figure 3:**
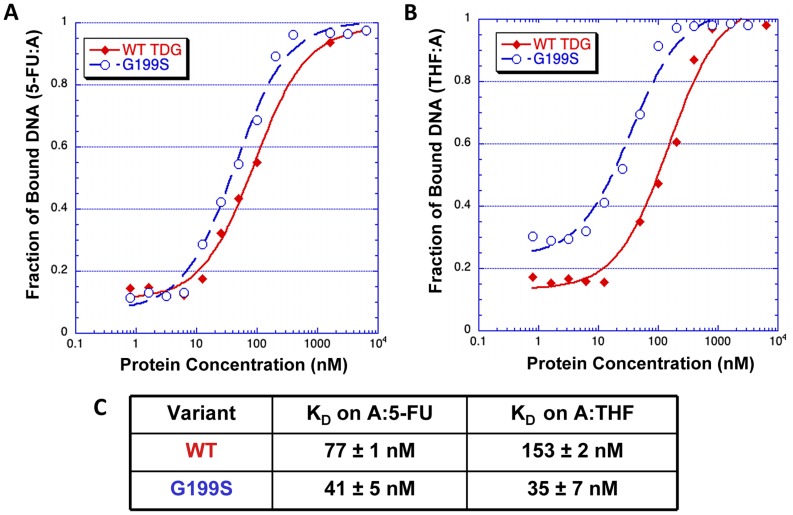
G199S has a slightly higher affinity for the 5-FU:A lesion and binds significantly more tightly to its abasic product. Increasing concentrations (0–5400 nM) of WT TDG (*filled diamonds*) or G199S (*open circles*) protein were incubated for 30 mins at room temperature with ^32^P-labeled oligonucleotides carrying a tetrahydrofuran moiety (THF) or 5-FU opposite template A (Table 1). Data were plotted as fraction of DNA bound *versus* TDG protein concentration to obtain the K_D_. A and B. The representative binding curve of WT TDG and G199S on 5-FU:A and THF:A, respectively. C. The dissociation constants (K_D_) for WT TDG and G199S on 5-FU:A and THF:A substrates.

The crystal structures of human TDG bound to DNA suggest (**Figure 2**) that Gly199 is an important residue for stabilizing the everted abasic site into the glycosylase active site pocket during lesion processing [9], [16]. Gly199 is located in a loop that approaches the DNA from the major groove. The glycine residue is within van der Waals distance of the abasic site product [9]. We hypothesized that alteration of amino acid residue 199 from a small, nonpolar glycine to a nucleophilic serine could affect product release by TDG since Gly199 is in close vicinity to the abasic site product formed after excision of the base. To test this hypothesis, we used purified protein in gel electromobility shift assays to characterize the ability of WT and G199S TDG to bind DNA containing either A:5-FU or tetrahydrofuran opposite template A (**Table 1**). G199S binds to its substrate slightly more tightly than WT (K_D_ = 77±13 nM and 41±5 nM, WT and G199S, respectively) (**Figure 3A and C**). Most importantly, G199S binds over 4-fold more tightly to DNA containing tetrahydrofuran (K_D_ = 153±33 nM and 35±7 nM, WT and G199S, respectively) (**Figure 3B and C**). These data show that G199S remains more tightly bound to its abasic product after base removal, which could prevent proper processing of the repair intermediate leading to DNA breaks and genomic instability.

### Accumulation and persistence of DNA single-strand and double-strand strand breaks in G199S-expressing cells after treatment with 5-FU

**Figure 4:**
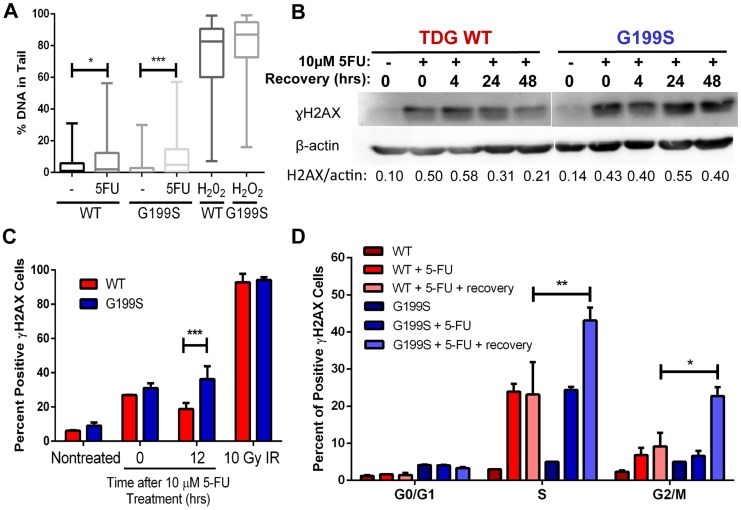
Accumulation of BER intermediates in G199S-expressing cells. A. MCF10A pools expressing WT or G199S were treated with 15 µM 5-FU for 24 hrs. Single-strand breaks (SSBs) were analyzed by the alkaline comet assay. H_2_O_2_ was used as a positive control. Shown are box plots with individual percentage of tail DNA per treatment group, medians, interquartile ranges (boxes), and maximum and minimum-percentiles (whiskers). At least 100 comets were scored per treatment group. * and *** denote p<0.05 and p<0.001, respectively. B. γH2AX western blot of MCF10A cells expressing WT or G199S treated with or without 10 µM 5-FU for 48 hrs and allowed to recover for up to 48 hrs in drug-free media. Quantification of γH2AX induction is below each lane. β-actin was used to normalize the amounts of protein in the extracts. C and D. MCF10A cells expressing WT or G199S were synchronized and treated with 10 µM 5-FU for 24 hrs and allowed to recover for 0 or 12 hrs. C. Cells were stained with γH2AX antibody to assess the levels of double-strand breaks (DSBs) and analyzed by flow cytometry. Data from two independent experiments are plotted as the mean ± SEM. *** denotes p<0.001. D. Cells were pulsed with BrdU before collection, fixed and stained with γH2AX antibody and propidium iodide to assess the levels of double-strand breaks (DSBs) per cell cycle phase and analyzed by flow cytometry. Data are graphed as mean ± SEM. ** and * denote p<0.01 and P<0.05, respectively.

Treatment of cells with the chemotherapeutic agent 5-fluorouracil (5-FU) leads to incorporation of 5-FU into DNA, removal of the 5-FU base by TDG, and DNA strand break accumulation that is dependent on TDG expression [17]. In addition, TDG is one of the major DNA glycosylases that recognizes and removes 5-FU incorporated opposite A *in vitro* and in cells [4], [17]. Therefore, we asked if treatment with 5-FU leads to the increased formation of BER intermediates in the human breast epithelial MCF10A cell line expressing G199S compared to WT-expressing cells. We generated stable MCF10A cell lines expressing HA-tagged TDG WT or G199S at equivalent levels in the WT endogenous background (**Figure S1**). Note that MCF10A cells themselves express WT TDG, so expression of G199S TDG in these cells mimics a heterozygous condition. We used the alkaline comet assay to quantify single-strand break (SSB) formation induced by 5-FU treatment of MCF10A cells expressing either TDG WT or the G199S variant. We observe a modest increase in SSBs in cells expressing WT after treatment for 24 hours with 5-FU, but a more significant increase in SSBs in cells expressing G199S TDG (**Figure 4A**) (p<0.05 versus p<0.001). These data suggest that base excision by TDG leads to DNA strand break formations but that there is greater accumulation of these breaks in cells expressing G199S TDG.

Next, we wished to determine if G199S induces increased levels of DNA double-strand breaks (DSBs) in cells treated with 5-FU using γH2AX as a marker of DSBs. We treated MCF10A cells expressing WT or G199S with 5-FU for 48 hrs followed by recovery in drug-free media for up to 48 hrs and analyzed γH2AX induction using western blot analysis. We show that while treatment with 5-FU induces DSBs in both WT and G199S-expressing cells as expected, expression of G199S leads to continued high levels of DSBs in cells after 24 hrs of recovery compared to WT cells (**Figure 4B**). In addition and more interestingly, these DSBs continue to persist in cells expressing G199S after 5-FU was removed from the media for up to 48 hrs, suggesting that there is a continuous formation of DSBs and/or a delay in repair of these BER intermediates. In order to further support these findings and to determine more specifically when these breaks were occurring during the cell cycle, we analyzed γH2AX staining using fluorescence-activated cell sorting (FACS). We synchronized MCF10A cells expressing WT or G199S by serum starvation and treated them as they were entering S-phase with 5-FU for 24 hrs followed by recovery in drug-free media for up to 12 hrs. We pulsed cells with bromodeoxyuridine (BrdU) for 30 mins prior to harvesting and stained with propidium iodide (PI) in order to determine cell cycle stage. In these experiments expression of G199S also leads to the accumulation of DSBs in cells treated with 5-FU (**Figure 4C**). As shown in **Figure 4D**, DSBs accumulate in G199S-expressing cells during S- and G2/M-phases of the cell cycle and continue to persist after 12 hrs of recovery, compared to WT-expressing cells where DSBs are more rapidly repaired. These data suggest that expression of G199S in cells treated with 5-FU results in the accumulation of DSBs. This may be a consequence of a replication fork collapse upon collision with an AP site or with an AP site bound to TDG.

### Expression of G199S delays S-phase progression and activates a DNA damage response to 5-FU

**Figure 5:**
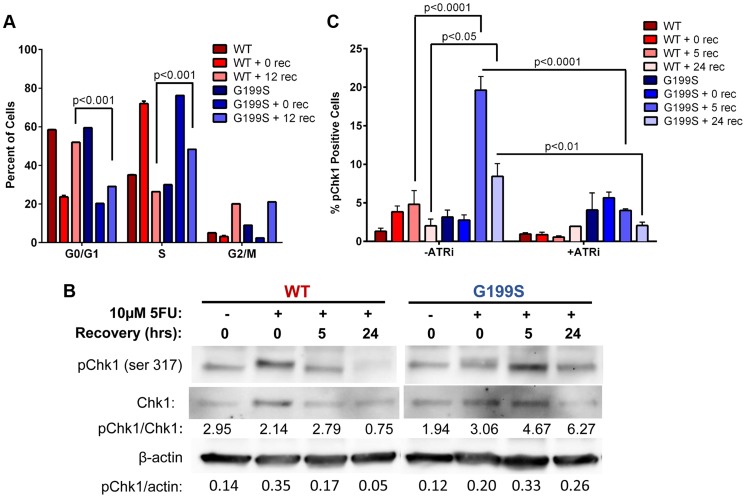
Expression of G199S delays S phase progression and activates a DNA damage response. A. MCF10A cells expressing WT or G199S were synchronized and treated with 10 µM 5-FU for 24 hrs and allowed to recover for 0 or 12 hrs. Cells were pulsed with BrdU before collection, fixed and stained with propidium iodide to determine the cell cycle phase and analyzed by flow cytometry. Data are graphed as mean ± SEM. B. MCF10A cells expressing WT or G199S were treated with or without 10 µM 5-FU for 24 hrs and allowed to recover for up to 24 hrs in drug-free media. Quantification of pChk1 induction is found below. Chk1 (middle panel) was used to normalize the amounts of protein in the extracts and β-actin (lowest panel) was used as a loading control. C. MCF10A cells expressing WT or G199S were treated with in the presence or absence of the ATR inhibitor VE-821 for 2 hrs prior to the addition of 10 µM 5-FU for 24 hrs and allowed to recover for up to 24 hrs. pChk1 status was analyzed by flow cytometry. Data are graphed as mean ± SEM.

It has been shown that Chk1 plays a crucial role in DNA damage and checkpoint control and is activated in HeLa and DT40 B-lymphoma cells in response to 5-FU treatment [18], [19]. These cells ultimately stall in S phase of the cell cycle due to accumulation of DSBs and slowing of DNA replication. Due to the fact that there are increased DSBs occurring in S- and G2/M-phases, we wished to determine if these result in the activation of a DNA damage response. Analysis of the cell cycle profile of MCF10A cells expressing WT or G199S after treatment with 5-FU shows that both WT and G199S-expressing cells initially stall in S-phase. After removal of the drug, WT-expressing cells begin recycling as indicated by the significant increase of cells in the G0/G1-phase 12 hours post recovery (**Figure 5A**). Conversely, G199S-expressing cells remain stalled in S-phase indicating that expression of G199S delays cell cycle progression in S-phase compared to WT. To determine if expression of G199S leads to an enhanced DNA damage response after treatment with 5-FU, we treated MCF10A cells expressing WT or G199S with 5-FU for 24 hrs followed by recovery for up to 24 hrs in drug-free media and analyzed phosphorylated Chk1 expression by western blot analysis (**Figure 5B**). Expression of G199S leads to prolonged induction of pChk1 in cells treated with 5-FU compared to WT. Chk1 phosphorylation is regulated by ataxia telangiectasia and Rad3 related (ATR) [20]. To test the requirement of ATR for G199S-mediated pChk1 induction after 5-FU treatment, we treated cells in the presence of absence of the ATR inhibitor VE-821 for 2 hrs before the addition of 5-FU for 24 hrs. Cells were allowed to recover for 0, 5, or 24 hrs and Chk1 phosphorylation was analyzed by flow cytometry. Cells expressing G199S had significant increases in pChk1 after 5 and 24 hrs recovery compared to WT cells under the same conditions (**Figure 5C**). Moreover, in the presence of the ATR inhibitor, the pChk1 levels were significantly attenuated suggesting that the activation of ATR is likely responsible for the phosphorylation of pChk1 at serine 345. Together, these results show that the DNA damage response is activated in G199S-expressing cells even after the drug has been removed, indicating that there is a delay in repair of 5-FU-induced lesions in the presence of G199S. This prolonged damage likely leads to genomic instability and could contribute to cellular transformation or increased drug sensitivity in these cells.

### Expression of G199S induces chromosomal aberrations

**Figure 6:**
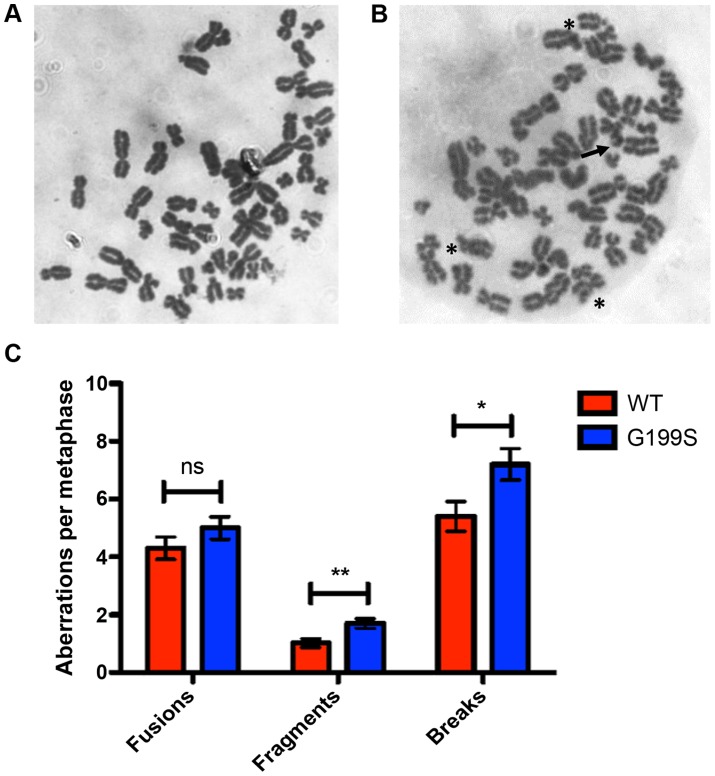
Expression of G199S leads to induction of chromosomal aberrations. Representative image of metaphase spreads of MCF10A pools expressing (A) WT or (B) G199S. Chromosomal breaks are noted with arrows and fusions are noted with *. C. Graph illustrating the average number of aberrations per metaphase. A total of at least 50 metaphases were scored for each cell line. Data are graphed as mean ± SD. ** and * denote p<0.01 and P<0.05, respectively.

Recent work has shown that expression of a germline variant of the n^th^ endonuclease III-like (NTH1) DNA glycosylase as well as several germline and tumor-associated variants of DNA polymerase β induce genomic instability in the form of chromosomal aberrations [21]–[24]. Given this, and the fact that G199S binds more tightly to its abasic product leading to activation of the DNA damage response and the accumulation of DSBs, we asked if expression of G199S in MCF10A human breast epithelial cells leads to the generation of chromosomal aberrations. Analysis of metaphase spreads shows significantly more breaks and fragments are present in G199S cells compared to WT (**Figure 6**). These data suggest that expression of G199S leads to increased levels of genomic instability in the form of chromosomal aberrations.

### Expression of G199S in human cells leads to cellular transformation

**Figure 7:**
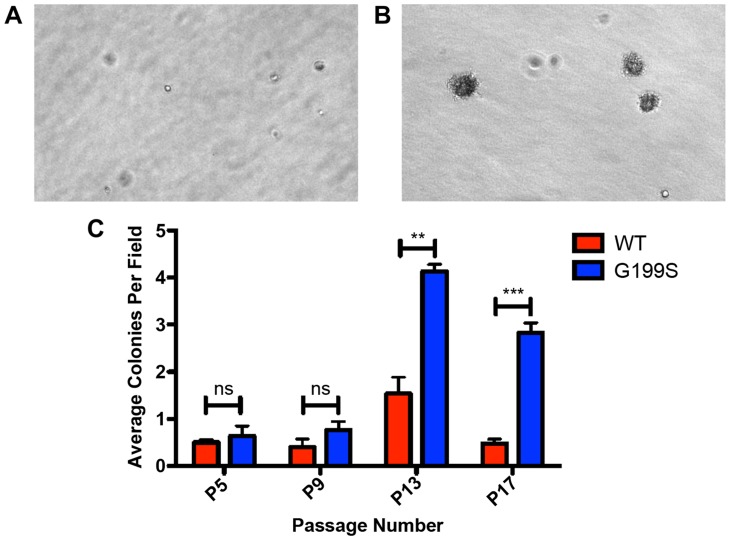
Expression of G199S induces cellular transformation. A. Representative image of anchorage-independent growth of MCF10A pools expressing WT TDG at passage 13 at 20× magnification. B. Representative image of anchorage-independent growth of MCF10A pools expressing G199S at passage 13 at 20× magnification. Note several large colonies have formed. C. The average number of colonies per field are plotted on the Y-axis. Cells were scored at passages 5, 9, 13 and 17 after 5 weeks of growth in soft agar. ** and *** denote p<0.01 and p<0.001, respectively.

Given that expression of G199S induces genomic instability, we tested the hypothesis that expression of G199S induces cellular transformation. We expressed HA-tagged TDG WT or G199S to equal levels in MCF10A pools and performed a soft agar growth assay in which cells that have transformed are able to grow in an anchorage-independent manner on soft agar, while non-transformed cells are unable to grow and will not form colonies. As shown in **Figure 7A–C**, cells expressing G199S form significantly more colonies at passage 13 as compared to WT, showing that expression of G199S induces a transformed phenotype. These data also suggest that individuals carrying the G199S variant are at an increased risk to develop cancer.

### Expression of G199S does not confer sensitivity to treatment with 5-FU

Although 5-FU is known to be an antimetabolite and to inhibit thymidylate synthase, 5-FU is also incorporated into DNA. It has recently been shown that TDG recognizes and removes 5-FU incorporated opposite A in cells [17]. Also, excision of 5-FU by TDG inhibits efficient downstream processing of lesions leading to the accumulation of BER intermediates, activation of a DNA damage response and eventual cell death as shown by us and others [17]. Because of the important role TDG plays in mediating 5-FU cytotoxicity and the fact that G199S has altered biochemical function and induced genomic instability in cells, we investigated whether expression of G199S would sensitize cells to 5-FU.

**Figure 8:**
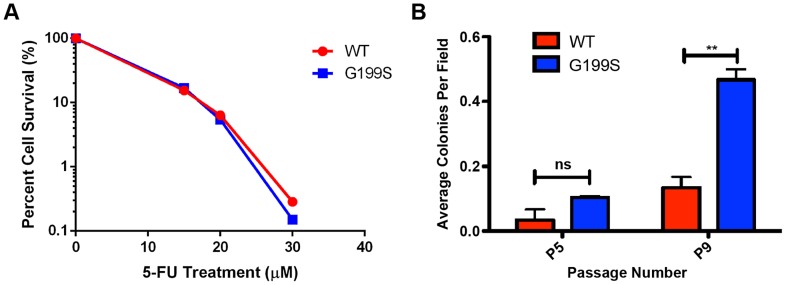
5-FU accelerates cellular transformation in G199S-expressing cells. A. Clonogenic survival assays were conducted with MCF10A pools expressing endogenous WT or G199S. Cells were treated with 0, 15, 20 or 30 µM 5-FU for 48 hrs, trypsinized and serial diluted on to plates. Cells were grown for 14 days, stained with crystal violet and colonies were counted. Representative image of data. Experiment was repeated three times and revealed the same trend. B. Anchorage-independent growth assay with MCF10A cells expressing WT or G199S after treatment with 1 µM 5-FU for 48 hrs. The average number of colonies per field are plotted on the Y-axis. Cells were scored at passages 5 and 9 after 5 weeks of growth in soft agar. ** denotes p<0.01.

We treated MCF10A cells expressing WT or G199S with increasing doses of 5-FU for 48 hrs and conducted a clonogenic survival assay. As shown in **Figure 8A**, expression of G199S did not sensitize cells to treatment with 5-FU. In combination with our chromosomal aberration studies, these data suggest that some cells expressing G199S that harbor genomic instability may survive and could become transformed.

### Cellular transformation is accelerated by treatment with 5-FU in cells expressing G199S

Due to the fact that cells expressing G199S are not more sensitive to treatment with 5-FU, we wished to determine the consequence of treating normal, non-transformed cells with a dose of 5-FU comparable to that used in the clinic. Cancer patients being treated with a 5-FU-based chemotherapy regimen are commonly treated with bolus or continuous infusion of 5-FU for up to 5 days [25]–[27]. Studies have shown that the plasma concentrations of 5-FU reach levels around 1–3 µM after administration of the drug [26]–[29]. We therefore decided to treated MCF10A cells expressing G199S or WT with a continuous dose of 1 µM 5-FU for 48 hrs followed by growth in drug-free media and monitor cellular transformation. As shown in **Figure 8B**, cells expressing G199S and treated with 5-FU exhibit significantly higher levels of anchorage-independent growth at passage 9 compared to cells expressing WT. Interestingly, treatment with 5-FU accelerated cellular transformation in G199S-expressing cells from passage 13 to passage 9 compared to non-treated cells (**Figures 7C** and **8B**). In combination, our results indicate that treatment of cells expressing G199S with 5-FU at a dose similar to that used in the clinic is enough to induce genomic instability leading to a transformed phenotype.

## Discussion

We provide evidence that in the presence of endogenous WT TDG, G199S acts in a dominant manner to induce genomic instability and leads to cellular transformation of human cells. The molecular basis of this phenotype resides in the ability of G199S TDG to bind more tightly to its abasic product than WT, leading to the accumulation of DNA strand breaks. Our findings are consistent with the interpretation that individuals harboring the rs4135113 SNP encoding G199S TDG may be at increased risk for cancer susceptibility.

### Slow turnover by G199S TDG leads to the formation of DNA strand breaks

Previous work has shown that TDG removes 5-FU from DNA and that this results in the accumulation of DNA strand breaks and checkpoint activation [17]. Significantly increased levels of strand breaks were observed in cells expressing TDG compared to TDG-deficient cells. TDG is released from its abasic product by a SUMO-dependent conformational alteration that results in decreased binding affinity for the abasic site [30], [31]. This slow release of TDG could lead to an accumulation of abasic sites that result in collapsed replication forks as the forks collide with AP sites or perhaps with TDG bound to an AP site. Our research supports and extends this work.

We show that expression of WT TDG leads to 5-FU-induced SSBs and DSBs. The SSBs most likely arise from the processing of BER intermediates. Concurring with the previous work, we suggest that the DSBs arise as a result of the collision of a replication fork with an abasic site or an abasic site bound to TDG itself. This suggestion is supported by our observation of the accumulation of cells in S-phase after treatment with 5-FU along with the ATR-dependent phosphorylation of Chk1, which is indicative of replication stress. Therefore, our work leads us to conclude that TDG is responsible for the removal of 5-FU that has been incorporated into DNA.

However, the major focus of our work is on the rs4135113 SNP, which encodes the G199S TDG protein. Although this protein exhibits similar catalytic efficiency in the removal of T opposite template G and 5-FU opposite template A, G199S TDG binds to its abasic product with significantly increased affinity compared to WT TDG. Based upon these *in vitro* results, we hypothesized that expression of G199S would lead to increased accumulation of DNA strand breaks in cells. We expressed G199S in MCF10A cells with WT TDG, but at equivalent levels as WT in an attempt to mimic the heterozygous condition observed in individuals harboring the rs4135113 SNP. Indeed, in cells expressing G199S, we observe accumulation of both SSBs and DSBs that is significantly increased over what we observe in cells expressing exclusively WT TDG. We postulate that these breaks accumulate as a result of the slow release of G199S from its abasic product due to tighter binding of the abasic site by Ser199 in comparison to Gly at that position, perhaps as a result of a structural distance of approach between the residue and the abasic site. The Cβ of serine is predicted to make a closer approach to the abasic ring, resulting in a stronger van der Waals interaction. In addition, the hydroxyl group of serine could hydrogen bond with the O4′ of the sugar moiety or the non-bridging oxygen of the adjacent phosphate, therefore further strengthening the interaction between TDG and the everted region of the DNA.

Interaction with SUMO has also been shown to be important for turnover of TDG. We feel that it is unlikely that alteration of Gly199 to Ser results in an aberrant interaction with SUMO, given our *in vitro* experiments demonstrating tight binding of G199S to is abasic product along with our observation that G199S appears to be sumoylated at levels that are fairly equivalent to what is observed with WT (**Figure S1**).

### G199S, genomic instability, and cellular transformation

TDG recognizes and removes several endogenous lesions, including T and U mispaired with template G, which arise due to spontaneous deamination events. In addition to this, two endogenous oxidation products of TET protein modification, 5-fC and 5-caC opposite G, are also removed by TDG [6], [7], [9]. Given that G199S removes DNA base damage that is produced endogenously, we surmised that expression of G199S in the MCF10A cells would lead to genomic instability. We showed that expression of G199S induces significantly higher levels of chromosomal aberrations that likely play an underlying role in the induction of cellular transformation. We suggest that WT and G199S TDG remove similar quantities of damaged bases. We imagine that when expressed in cells, WT TDG is able to remove the damaged base and turnover efficiently, allowing BER to be completed. G199S, however, exhibits higher affinity for its abasic product and likely prevents proper downstream processing of the lesion. If cells enter S-phase and the abasic lesion has not been resolved, the abasic site itself or an abasic site with G199S protein bound to it would block replication fork progression. There will be initial stabilization of the replisome, but if G199S is not removed and the lesion is not repaired efficiently, there will be fork destabilization, strand breakage and eventual formation of DSBs. If the level of breaks is high enough in these cells and homology-directed repair cannot correct the damage, this could result in genomic instability as a result of the repair of DNA strand breaks by nonhomologous end-joining and, ultimately, lead to cellular transformation.

In this study, we focused our attention on lesions opposite A due to the fact that 5-FU is predominantly incorporated opposite A. It has long been established though that TDG preferentially binds to and processes lesions with template G due to the specific hydrogen bonds formed between TDG's catalytic core and the template G [32]. In fact, previous work has demonstrated that TDG exhibits extremely tight binding to an AP site opposite G, K_D_ = 1.4 nM and 6.2 nM, depending on the substrate used [33]. Our findings that expression of G199S spontaneously induced chromosomal aberrations and leads to genomic instability suggests that G199S also binds more tightly to AP sites opposite G.

### 5-FU treatment of cells with TDG G199S decreases the latency of cellular transformation

Our results showing increased accumulation of DNA strand breaks after treatment of cells harboring G199S versus WT with 5-FU suggested that G199S-expressing cells might be sensitive to treatment with 5-FU. This was not the case and MCF10A cells expressing WT alone or in combination with G199S exhibited similar sensitivity to 5-FU. Given this result, we speculated that treatment of G199S-expressing cells with 5-FU could lead to cellular transformation as a result of aberrant repair of high levels of DSBs. We showed that treatment of cells expressing G199S with 5-FU at doses comparable to those in the clinic leads to a decreased latency of cellular transformation compared to cells not treated with 5-FU and also compared to cells expressing WT, both in the presence and absence of 5-FU. These results are consistent with the idea that treatment of individuals harboring the G199S TDG germline variant with 5-FU could drive carcinogenesis. Thus, germline DNA repair variants have the capacity to impact treatment outcomes.

## Materials and Methods

### Materials

All oligonucleotides used for the *in vitro* biochemical assays were purchased from Keck Biotechnology Resource Laboratory at Yale University and purified by denaturing polyacrylamide gel electrophoresis. [γ-^32^P]ATP (5 mCi) and ATP were purchased from PerkinElmer Life Sciences and Sigma, respectively. T4 polynucleotide kinase was purchased from New England Biolabs. 5-fluorouracil was purchased from Sigma. The ATR inhibitor VE-821 was purchased from Selleckchem.

### Plasmids and cloning

The TDG variant G199S was generated using the Stratagene QuikChange site-directed mutagenesis kit according to manufacturer's instructions. The primers used for these reactions were purchased from Invitrogen and the sequences are available upon request. For protein expression and biochemical studies, the pET28a plasmid containing WT TDG and an N-terminal His-tag was used as a template. For cell culture experiments, the pRVYtet retroviral vector containing WT TDG and a C-terminal hemaglutinin (HA)-tag was used. The sequence of positive clones were generated at the Keck DNA Sequencing Facility at Yale University.

### Protein expression and purification

The N-terminally 6×His-tagged human WT and G199S TDG cloned in a pET28a vector were expressed in the *Escherichia coli* strain BL21 (DE3)-competent cells. Fifty milliliters of seed cultures were inoculated in 2 LB (20 mg/ml tryptone, 10 mg/ml yeast extract, 10 mg/ml NaCl) containing 100 mg/ml of kanamycin, and grown at 37°C to OD600: 0.8, 0.4 mM IPTG was added to induce protein expression and the culture was then incubated at 16°C overnight. Cultures were pelleted and frozen. Frozen pellets were resuspended in 8 mL of cell lysis buffer (50 mM Tris–HCl (pH 7.5), 10% sucrose, 10 mM EDTA, 600 mM KCl, 1 mM dithiothreitol, 0.01% Igepal CA-630 (Sigma-Aldrich, St. Louis, MO)) for 1 g of cell pellet, in the presence of protein inhibitors (chymostatin, leupeptin, aprotinin, and pepstatin, at 2 mg/ml each, 1 mM phenylmethanesulfonyl fluoride) and sonicated at 50% power 10 times 30 s with 20 s rest between each pulse. The cell lysate was ultracentrifuged at 45 k rpm for 1 h and the clarified supernatant was incubated with 10 mM imidazole and 1.5 ml Ni-NTA agarose (Qiagen) for 2 h at 4°C. Beads were washed with buffer K (20 mM KH_2_PO_4_, pH 7.4, 0.5 mM EDTA, 10% glycerol, 0.01% Igepal CA-630) supplemented with 1 M KCl, 10 mM imidazole, 1 mM ATP, and 8 mM MgCl_2_, and then washed with buffer K supplemented with and 10 mM imidazole. Nickel-bound protein was eluted with buffer K containing 200 mM imidazole and 150 mM KCl. Eluates were pooled and resolved on a 10 mL SP sepharose fast flow (GE Healthcare) column using an AKTA FPLC (GE healthcare) with a 20 column volume gradient of 50 to 800 mM KCl in buffer K. Fractions containing TDG were pooled, loaded and resolved on a 6 mL MHAP column with a 20 columns volume gradient of 0 to 300 mM KH_2_PO_4_ in K buffer. Fractions were identified on SDS page gels, pooled and the protein was concentrated using a 30 kDa cutoff Amicon ultra centrifugal device (Millipore) to a volume of 250 µL and loaded on a Superose 6 10/300 GL size exclusion chromatography column (GE healthcare) run with K buffer containing 300 mM KCl. Fractions containing TDG to near homogeneity were pooled, concentrated and the buffer exchanged to a storage buffer (20 mM Tris–HCl pH 7.5, 100 mM KCl, 0.5 mM EDTA, 5 mM β-mercaptoethanol and 30% glycerol). The protein was then aliquoted and stored at −80°C.

### Preparation of DNA substrates

Oligonucleotides (28- and 38-mers) were synthesized by the Keck Biotechnology Resource Laboratory at Yale University and purified by denaturing polyacrylamide gel electrophoresis prior to use. The template oligonucleotide was labeled at the 5′ end using T4 polylnucleotide kinase and [*γ*-^32^P]ATP. Kinased oligonucleotides were purified using Microspin columns (Bio-Rad) to remove any unincorporated label and annealed to unlabeled complementary oligonucleotides at a 1∶1.2 ratio in 150 mM NaCl, 10 mM Tris-HCl (pH 8.0) and 1 mM EDTA. The mixtures were heated to 95°C for 5 mins and slowly cooled to room temperature for 2–3 hrs. The annealing was verified using a 12% native polyacrylamide gel followed by autoradiography.

### Single turnover assay

Kinetic assays were performed using radiolabeled double-stranded 38-mer oligonucleotides containing either T opposite G or 5-FU opposite A. Purified WT TDG or G199S protein (75 nM) was incubated with 5 nM DNA substrate at 37°C for up to 20 mins in 1× nicking buffer [20 mM HEPES pH 7.5, 1 mM EDTA, 1 mM DTT and 1 mg/ml BSA]. At the indicated time points, an aliquot of the reaction was treated with 0.5 M NaOH and incubated at 90°C for 30 mins to cleave the abasic site. After the addition of denaturing gel loading buffer [95% formamide, 20 mM EDTA, trace Bromophenol blue), substrate and product were resolved using denaturing 20% PAGE and visualized and quantitated using a Storm 860 phosphorimager with ImageQuant software. The substrate and product bands were quantified to evaluate the percentage of substrate converted into product. Data from the single-turnover experiments were then plotted and fit to a first-order rate equation using nonlinear least-squares analyses with Kaleidagraph software (Synergy software). Catalytic rates (*k_st_*) were calculated as the mean of two separate experiments with error bars representing ±1 SD.

### Gel electrophoretic mobility shift assay

Increasing concentrations of WT TDG or G199S protein (0–6400 nM) were incubated with 2 nM ^32^P-labeled 28-mer oligonucleotides carrying 5-FU or a tetrahydrofuran nucleotide (THF) opposite A for 30 mins at RT in binding buffer [20 mM HEPES (pH 7.3), 1 mM EDTA, 10 mM (NH_4_)_2_SO_4_, 1 mM DTT, 0.2% Tween-20, 30 mM KCl]. The reactions were mixed with loading buffer [60% 0.25× TBE, 40% Glycerol, trace Bromophenol blue] and immediately loaded onto a 6% Native PAGE gel [0.5× TBE, 5% Glycerol] while the current was running at 150 V. This gel had been prerun at 150 V for 1 hr at 4°C. Once the samples had left the wells, the gel was run for approximately 3 hrs at 150 V at 4°C, dried and exposed in a phosphorimager cassette for 24–72 hrs. The data were visualized and quantified using a phosphorimager and ImageQuant software. To estimate the apparent K_D_ for each enzyme on the DNA substrate, the percent bound DNA was plotted vs the log of [DNA] in nM, and the data was fit to the following equation using Kaleidagraph:
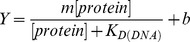
where *Y* is the amount of bound protein, *m* is a scaling factor, and *b* is the apparent minimum *Y* value. Dissociation constants were calculated as the mean of three separate experiments with error bars representing ±1 SD.

### Cell lines and cell culture

MCF10A cells are immortalized, nontransformed epithelial cells derived from human mammary tissue (ATCC). These cells were maintained in DMEM/F12 medium (Invitrogen) supplemented with 5% horse serum (Invitrogen), 1% penicillin-streptomycin, epidermal growth factor (20 ng/ml), hydrocortisone (0.5 mg/ml), cholera toxin (100 ng/ml), insulin (10 mg/ml) (Sigma-Aldrich) and grown at 37°C in a 5% CO_2_ humidified incubator. Stably expressing MCF10A lines were selected by adding 250 mg/ml hygromycin B (Invitrogen) to the media. The GP2-293 virus packaging cell line (Clontech) used for retrovirus preparation was maintained in DMEM (Invitrogen) supplemented with 10% fetal bovine serum (Invitrogen), 1% L-glutamine (Invitrogen), 1% penicillin-streptomycin (Invitrogen) and 1% HEPES (Invitrogen).

### Transfection, infection and expression analysis

Human TDG WT and G199S constructs were packaged into retrovirus using the GP2-293 packaging line. The pRVYtet constructs were cotransfected with the pVSV-G plasmid (10 µg of each) into GP2-293 cells using standard calcium phosphate transfection, allowed to grow for 72 hrs and retrovirus was harvested. To generate stably-expressing pools, MCF10A cells were grown to approximately 30% confluence and infected with fresh retrovirus in the presence of 4 mg/ml polybrene (Sigma) for 3 hrs. Cells were incubated overnight in fresh media in the presence of 4 mg/ml polybrene. For selection of pools, cells were split 1∶3 the day after infection and 250 mg/ml hygromycin B was added to the media 24 hrs later.

Expression of exogenous HA-tagged WT and G199S was verified by Western blot. Cells at 80–90% confluence were harvested by scraping into lysis buffer [10 mM Tris (pH 7.4), 1 mM EDTA, 0.1% SDS, 180 µg/ml PMSF] and sonicated for 15 sec. at 10% amplitude. Protein concentrations were determined using the DC Protein Assay (BioRad) and equal amounts of protein were mixed with sample buffer and boiled for 5 min. The samples were resolved on a 10% SDS-polyacrylamide gel and transferred to a nitrocellulose membrane. TDG was detected by incubating the membrane using anti-HA antibody (Covance, MMS-101P) at 1∶600. DNA damage was assessed by Western Blot using anti-γH2AX (Millipore, 05-636) at 1∶750. An antiserum raised against actin (Sigma, A5441) was used to quantify this protein, which served as a loading control. Blots were imaged using Bio Rad ChemiDoc XRS+ and quantified using Image Lab Software.

### Cellular transformation

Anchorage-independent growth was assessed as previously described [21]. Briefly, a total of 1×10^4^ MCF10A cells were mixed with media containing 0.7% noble agar (USB) at 42°C. This mixture was poured onto a layer of media containing 1.0% noble agar in a well of a 6-well dish. Cells were grown at 37°C in a 5% CO_2_ humidified incubator and fed twice weekly. The number of colonies present in each of 10 microscope fields per well from a total of 3 wells per experiment were counted after 5 weeks of growth.

### Metaphase spreads

Metaphase spreads were prepared and chromosomal aberrations were analyzed as described previously [22]. Briefly, TDG WT and G199S-expressing MCF10A cells were seeded in 10 cm dishes. 100 ng/ml colcemid (Invitrogen) was added for 5 hrs before harvesting. Cells were trypsinized, washed one time with PBS and centrifuged at 100 g for 5 mins at RT to pellet the cells. Cells were resuspended dropwise in hypotonic solution [75 mM KCl] for 30 mins at 37°C. Cells were then fixed by gradual resuspension in Carnoy's Fixative [75% methanol, 25% acetic acid] for 15 mins. Finally, cells were dropped onto microscope slides, dried and stained with Giemsa. Well-spread metaphases were identified under 100× objective (Zeiss) and images were taken using Spot Camera software (Diagnostic Instruments). Metaphase spreads were blindly scored for chromosomal fusions, breaks and fragments.

### Single-cell gel electrophoresis (alkaline comet assay)

Equal numbers of cells (5×10^6^) cells were plated in 10 cm dishes. The following day or the day after, cells were treated with 15 µM 5-FU for 24 hrs and allowed to recover for 0 or 24 hrs in drug-free media. After treatment, the cells were prepared and analyzed immediately according to published procedures (Trevigen). Image analysis of 100 cells was performed using CometScore software (TriTek, Sumerduck, VA). Data are represented as means ± SEM.

### Western blot analysis to assess DNA damage response

HA-tagged WT or G199S were expressed in MCF10A cells and expression was verified as describe above. 500,000 cells were plated in 10 cm dishes and treated with 10 µM 5-FU for 24 or 48 hrs followed by recovery up to 12 or 24 hrs in drug-free media. Cells were washed one time with PBS and harvested by scraping into PBS before being spun down and frozen at −80°C. Cells were thawed on ice and lysed in AZ buffer [50 mM Tris (pH 8), 250 mM NaCl, 1 mM EDTA, 0.1% Igepal, 0.1% SDS, 10 mM Na_4_P_2_O_7_, 10 mM NaF, 1 mM PMSF, 1× PhosStop (Roche), 1× complete protease inhibitor (Roche)] on ice for 15 mins and vortexed every 5 mins. Samples were spun down and the supernatant was transferred to a new tube. Protein concentrations were determined using the DC Protein Assay (BioRad) and equal amounts of protein were mixed with sample buffer and boiled for 5 min. The samples were resolved on a 4–20% SDS-polyacrylamide gel and transferred to a PVDF membrane. γH2AX and pChk1 were detected by Western Blot using anti-γH2AX (Millipore, 05-636) at 1∶750, anti-pChk1 (Cell Signaling, #2344) at 1∶500 and anti-Chk1 (Cell Signaling, #2360) at 1∶750. An antiserum raised against actin (Sigma, A5441) was used to quantify this protein, which served as a loading control. Blots were imaged using Bio Rad ChemiDoc XRS+ and quantified using Image Lab Software.

### Flow cytometry

MCF10A cells expressing WT or G199S were seeded at 4×10^5^ cells per 10 cm dish and allowed to attach overnight. Cells were then synchronized by serum and growth factor starvation for 24 hrs. Complete media was added back and cells were further grown for 16 hrs at 37°C/5% CO_2_ to reach S phase. Cells were untreated or treated with 10 µM 5-FU for 24 hrs and allowed to recover in drug-free media for 0, 12 or 24 hrs after treatment. Cells were pulse labeled with 10 µM of 5-Bromo-2′-deoxyuridine (BrdU) (Sigma) for 30 mins at 37°C before cells were collected. Cells were washed once with PBS, pelleted and resuspended by adding 70% ice-cold ethanol dropwise while vortexing. The cells were fixed overnight at −20°C. The cells were centrifuged, the ethanol was aspirated and the cells were resuspended in 2 N HCl/Triton x-100 to denature the DNA and incubated for 30 mins at RT. Cells were then resuspended in 0.1 M Na_2_B_4_O_7_ (pH 8.5) to neutralize the sample. Cells were washed twice with BSA-T-PBS [1% Bovine Serum Albumin, 0.2% Triton X-100 in PBS]. Cells were incubated with anti-BrdU-FITC (Becton Dickinson) and anti-phospho-histone H2AX (Ser139) rabbit antibody (Cell Signaling) at 1∶200 overnight in the dark at 4°C. Cells were washed twice and incubated with anti-rabbit Alexa647 (Molecular probes, Invitrogen) at 1∶100 for 1 hr at RT in the dark. Cells were washed and resuspended in PI/RNase staining buffer (BD Pharmigen). For pChk1 analysis, cells were incubated with anti-phospho Chk1 (Ser345) (Cell Signaling, #2341) rabbit antibody at 1∶200 overnight followed by an incubation with anti-rabbit Alexa 647. Cells were resuspended in PBS. Fluorescence was analyzed by flow cytometry using the BD FACSCalibur and analyzed using FlowJo 8.8.6 software.

### Statistics

Two-tailed t tests and two-way analysis of variant (ANOVA) were used as appropriate. Sidak's multiple comparisons test was used to determine significant differences between means of each group. All statistics were performed using GraphPad Prism version 6 (GraphPad Software, San Diego). Data are represented as means ± SEM.

## Supporting Information

Figure S1 **Equivalent expression of recombinant HA-tagged TDG WT and G199S.** In the upper panel of the Figure, the lower band is HA-tagged TDG while upper band is HA-tagged TDG modified by SUMO conjugation. β-actin (lower panel) was used as a loading control. Quantification of TDG expression by normalizing to β-actin is listed below the image. Exogenous WT and G199S are expressed at equal levels.
(TIF)
