# Supplementary figures and images for: A Germline Polymorphism of Thymine DNA Glycosylase Induces Genomic Instability and Cellular Transformation
Source: PLoS Genet. 2014 Nov 6;10(11):e1004753. doi: 10.1371/journal.pgen.1004753 (PMC4222680; doi:10.1371/journal.pgen.1004753)

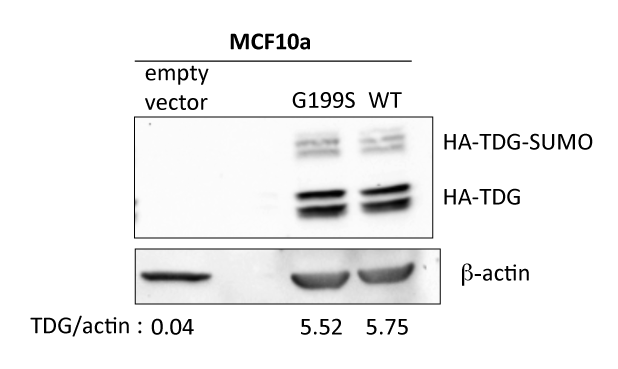

Supplement: Figure S1 — Equivalent expression of recombinant HA-tagged TDG WT and G199S. In the upper panel of the Figure, the lower band is HA-tagged TDG while upper band is HA-tagged TDG modified by SUMO conjugation. β-actin (lower panel) was used as a loading control. Quantification of TDG expression by normalizing to β-actin is listed below the image. Exogenous WT and G199S are expressed at equal levels. (TIF) [file pgen.1004753.s001.tif]
